# Thermal Conductivity of AlSi10MnMg Alloy in Relation to Casting Technology and Heat Treatment Method

**DOI:** 10.3390/ma17215329

**Published:** 2024-10-31

**Authors:** Iva Nováková, Milan Jelínek, Martin Švec

**Affiliations:** Department of Engineering Technology, Faculty of Mechanical Engineering, Technical University of Liberec, Studentská 1402/2, 461 17 Liberec, Czech Republic; iva.novakova@tul.cz (I.N.); milan.jelinek@tul.cz (M.J.)

**Keywords:** aluminium alloy, thermal diffusivity, thermal conductivity, casting, heat treatment

## Abstract

Nowadays, with the development of electromobility, the requirements not only for the mechanical properties but also for the thermal conductivity of castings are increasing. This paper investigates the influence of casting and heat treatment technology on the thermal diffusivity and thermal conductivity of an AlSi10MnMg alloy. The thermal diffusivity was monitored as a function of temperature in the range of 50–300 °C for the material cast by high-pressure die casting (HPDC) and also by gravity sand casting (GSC) and gravity die casting (GDC). This study also investigated the effect of the T5 heat treatment temperature (artificial ageing without prior solution treatment—HT200, HT300, and HT400) on the thermal conductivity of the material cast by different technologies. Experiments confirmed that the thermal diffusivity or thermal conductivity of the alloy depends on the casting technology. The slower the cooling rate of the casting, the higher the thermal conductivity value. For the alloy in the as-cast condition, the thermal conductivity at 50 °C is in the range of about 125 to 138 [W·m^−1^·K^−1^]. Regardless of the casting method, the thermal conductivity tends to increase with temperature (50–300 °C). Furthermore, a positive effect of heat treatment without prior solution treatment (HT200, HT300, and HT400) on the thermal conductivity was demonstrated. Regardless of the casting method of the samples, the thermal conductivity also increases with increasing heat treatment temperature. The results further showed that when artificial ageing is performed in industrial practice on castings to increase mechanical properties in the temperature range of 160–230 °C, this heat treatment has a positive effect on thermal conductivity.

## 1. Introduction

In industrial practice, aluminium alloy parts, mainly for the automotive and electronics industries, are produced using high-pressure die casting technology. This technology makes it possible to produce thin-walled moulded parts. However, the porosity of the castings makes it impossible to carry out a subsequent T6 or T7 heat treatment (solution treatment followed by ageing) to improve their mechanical properties. For parts produced by standard high-pressure die casting technology, only T5 heat treatment (artificial ageing without prior solution treatment) can be carried out. Parts requiring T6 or T7 heat treatment to achieve high mechanical properties are therefore cast under high vacuum.

With the development of electromobility, parts such as motor housings, heat sinks, battery trays, and inverters are required to have not only mechanical properties, but also thermal conductivity. The current requirements of the industry lead to the need to study the thermal conductivity of aluminium alloys that also meet the requirements for mechanical properties. The AlSi10MnMg alloy studied is one of these alloys.

The structure of the Al-Si-based hypoeutectic alloy is formed by a solid solution of α(Al) and eutectic (α(Al) + Si). The chemical composition of the intermetallic phases in Al-Si-based alloys depends on the presence and amount of each element [1].

A Si content of approximately 10% gives the alloy excellent castability. The alloy contains Sr to ensure a uniform distribution of the eutectic in the structure in the as-cast state. The strength and ductility properties of these alloys depend on the Mg content. In the as-cast state, the Mg forms the intermetallic phase Mg_2_Si which forms the Al-Si-Mg_2_Si eutectic. In the as-cast state, Mg has little effect on strength but reduces ductility. When the alloy is heat-treated, the strength properties increase with higher Mg content. These alloys also have a limited Fe content, although Fe has a beneficial effect on die soldering. In aluminium alloys, Fe at low levels forms several intermetallic phases which reduce strength properties, particularly ductility. The least favourable is the needle-shaped β-Al-Fe-Si (Al_5_FeSi) phase. In general, efforts are made to eliminate the adverse effect of Fe with the addition of Mn. The Mn content should be approximately half the Fe content. The literature refers to the Mn content not exceeding 0.2% as it adversely affects ductility, but tests have shown that to maximise ductility the Mn content should be in the range of 0.5–0.8%. At this level, the Mn content has a favourable effect on die soldering. The structure then contains α-AlFeMnSi (Al_15_(Fe, Mn)_3_Si_2_) particles, which can have different morphologies, ranging from Chinese script to polyhedral [2,3]. The authors [4] report that in addition to α-AlFeMnSi particles, a tetragonal δ-phase (Al_4_(FeMn)Si_2_) occurs in the structure, which can contain Mn in a wide range and is morphologically indistinguishable from the needle-like β-Al-Fe-Si phase.

Many authors have studied the thermal conductivity of aluminium alloys. Many parameters influence the thermal conductivity. Temperature is one of the critical parameters affecting the thermal conductivity of alloys [5,6]. Another is the chemical composition of the alloy and its structure. The literature indicates that the higher the strength properties of Al-Si alloys in the as-cast state, the lower the thermal conductivity [7,8,9]. This is because all methods of hardening the alloys (mechanical hardening, solid solution hardening, precipitation hardening, etc.) have a negative effect [5,10].

Alloying elements added to aluminium increase its strength but reduce its thermal conductivity [5,11]. The amount of alloying elements in a solid solution is limited. If the alloying element content exceeds its solid solubility limit, the excess will be present in the form of precipitated secondary phases [10,12,13,14].

For example, Si may be present in the Al-Si alloy in a solid solution and as a secondary phase. The thermal conductivity of Al-Si alloys is also influenced by the type of eutectic or its morphology [15,16,17]. Modification of the eutectic by strontium and changing its morphology from lamellar to fibrous have a favourable effect on conductivity [18,19,20].

Alloying elements dissolved in a solid solution have a greater effect on reducing thermal conductivity than precipitated elements. Of the elements dissolved in a solid solution, Zn, Cu, Mg, Si, and Mn have the least effect on thermal conductivity [11]. Because alloys contain multiple alloying elements that interact with each other, the theory of thermal conduction of metals cannot quantify their influence on conductivity.

Whether alloying elements in aluminium alloys dissolve in the matrix or exist as a secondary phase, they inhibit the movement of dislocations, improving mechanical properties, while scattering electrons and reducing thermal conductivity [5].

The thermal conductivity of aluminium casting alloys is also influenced by the casting technology. The casting process and its technological parameters determine the resulting structure and porosity of the casting [5,21,22]. Chen et al. [23], who investigated the thermal conductivity of a gravity-cast Al-Si-Cu-Fe-Zn alloy, reported that the gravity-cast alloy has higher thermal conductivity than die-cast alloys. The effect of porosity of A380 alloy was investigated by Ramirez et al. [24], who reported that the thermal conductivity decreases slightly with increasing porosity.

The thermal conductivity of castings is also affected by heat treatment [25,26,27,28]. Lumley et al. [29] showed that two-stage heat-treated (T4 and T6) Al-Si alloys have finer and more spherical eutectic Si particles and higher thermal conductivity than the alloys in the as-cast condition. These processes affect the thermal conductivity of aluminium alloys by changing the existing states of the alloying elements and the morphology of the secondary phases.

Current industrial practice places great emphasis on the level of thermal conductivity of the produced castings and is usually prescribed by the production regulation. The aim of this article was to provide specific thermal diffusivity (thermal conductivity) values for one of the most used alloys in present industry practice and to show the effect of different casting methods and heat treatment on the changes in thermal diffusivity. The presented data could be helpful for a number of companies to solve problems with insufficient or, on the contrary, too much thermal conductivity of their products.

## 2. Materials and Methods

In this paper, the thermal conductivity of the AlSi10MgMn alloy has been investigated. The chemical composition of the alloy was determined using a Q4 TASMAN optical spectrometer (Bruker Elemental GmbH, Berlin, Germany) and is shown in Table 1.

Samples for thermal conductivity measurements were taken from the castings. To observe the effect of casting technology on the thermal conductivity of the material, samples were taken from gravity sand casting (GSC), gravity die casting (GDC) and high-pressure die casting (HPDC). The HPDC samples were supplied by an industrial partner. They were from a structural part produced under high vacuum. The gravity castings were made in the laboratory. The material was melted, cleaned with refining salt, and then cast at a temperature of 720 °C. A casting with a diameter of 16 mm and a length of 100 mm was produced by gravity casting.

To investigate the effect of heat treatment (artificial ageing), the samples were further treated at 200 °C, 300 °C, and 400 °C without prior solution treatment (HT200, HT300, and HT400). The samples were heat-treated in an Elsklo MF3 furnace (Elsklo, Desna v J. h., Czech Republic). The holding time at the artificial ageing temperature was 1 h, followed by air cooling.

The thermal diffusivity of the samples was measured using a DLF 2 (Discovery laser flash, TA Instruments, New Castle, UK). The test specimens, with a diameter of 12.68 mm and a thickness of 4 mm, were machined from castings. The input parameter for the diffusivity measurements is the temperature dependence of the material density. This dependence was determined by calculation based on the coefficient of thermal expansion measured using a DIL 805L dilatometer (TA Instruments, New Castle, UK). The samples used to determine the coefficient of thermal expansion were 4 mm in diameter and 10 mm in length. The specific heat capacity was measured during the thermal diffusivity measurement of each sample by comparison with a molybdenum standard. The thermal conductivity λ was then determined by calculation. The diffusivity was monitored as a function of temperature over the range of 50–300 °C.

The microstructure of the samples was studied on a Tescan Mira 3 electron microscope (Tescan Orsay Holding a.s., Brno, Czech Republic) equipped with an Oxford UltimMax65 energy dispersive detector (Oxford Instruments plc, Oxfordshire, UK) for local chemical analysis. Grain size and orientation were analysed using an Oxford SYMMETRY detector (Oxford Instruments plc, Oxfordshire, UK).

## 3. Results

### 3.1. Structure of the Studied Alloys

Cooling conditions affect the formation and distribution of secondary phases. The structure of the Al-Si-based hypoeutectic alloy consists of a solid solution of α(Al), a eutectic (α(Al) + Si), and other secondary particles which vary depending on the added alloying elements.

**Figure 1:**
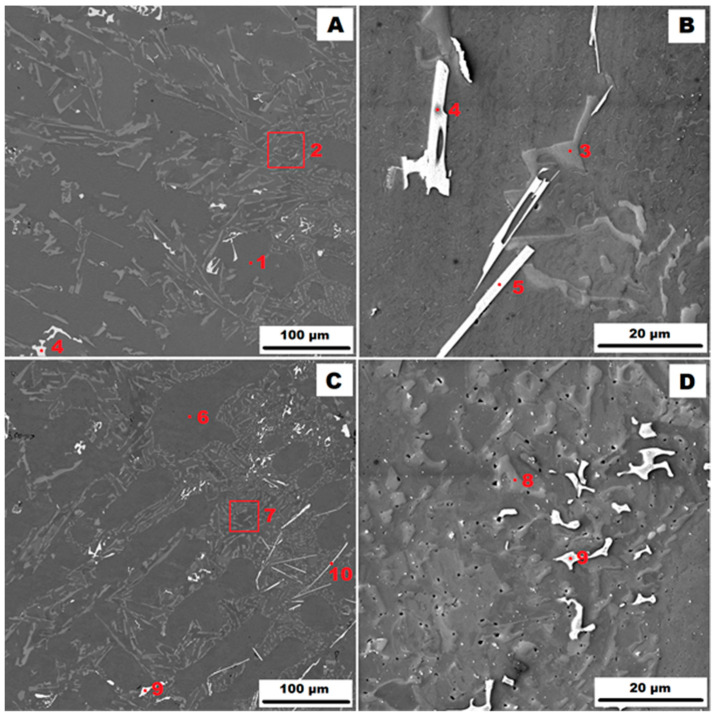
Structure of the AlSi10MnMg alloy cast into a sand mould (GSC): (**A**)—as-cast condition (HV 10 kV, BSE); (**B**)—as-cast condition (HV 10 kV, SE); (**C**)–condition after HT400 (HV 10 kV, BSE); and (**D**)—condition after HT400 (HV 10 kV, SE).

The slowest heat dissipation can be expected when casting into a sand mould (GSC). The structure of the AlSi10MnMg alloy cast into a sand mould in the as-cast state is shown in Figure 1A,B and the state after heat treatment at 400 °C for 1 h (HT400) in Figure 1C,D. The structure consists of a solid solution of α(Al)—points 1 and 6 in Figure 1—and a lamellar eutectic (α(Al) + Si)—points 2, 3, 7, and 8 in Figure 1. When slowly cooled in a sand mould, the eutectic is coarse. The structure also contains secondary α-AlFeMnSi particles, i.e., Al_15_(Fe, Mn)_3_Si_2_—points 4 and 9 in Figure 1—which are distributed in the form of individual sharply defined particles. In addition, the β-AlFeSi phase, i.e., Al_5_FeSi, which has the shape of long needles, is commonly found in the structure of Al-Si-based alloys. In the case of the studied AlSi10MnMg alloy, the β phase has been replaced by a more rarefied δ phase, i.e., Al_4_(Fe, Mn)Si_2_, due to the addition of Mn—see points 5 and 10 in Figure 1. Similar to the β-phase, the δ-phase was also present as long sharp needles, which is unfavourable from the point of view of mechanical properties because the brittle δ-phase needles can act as crack initiators. The identification of all phases present by EDX analysis is summarised in Table 2. Comparison of Figure 1A–D shows that there were no significant changes in the structure after heat treatment.

**Figure 2:**
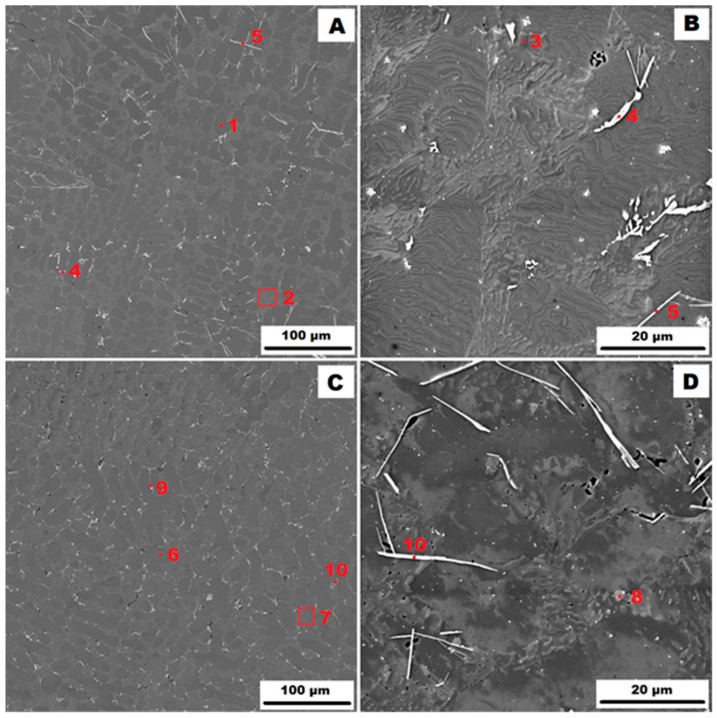
Structure of gravity die-cast (GDC) AlSi10MnMg alloy: (**A**)—as-cast condition (HV 10 kV, BSE); (**B**)—as-cast condition (HV 10 kV, SE); (**C**)—condition after HT400 (HV 10 kV, BSE); and (**D**)—condition after HT400 (HV 10 kV, SE).

The microstructure of the gravity die-cast (GDC) AlSi10MnMg alloy is shown in Figure 2. The as-cast condition is shown in Figure 2A,B and the condition after heat treatment at 400 °C for 1 h (HT400) is shown in Figure 2C,D). It can be seen that the higher cooling rate of the metal mould (GDC) compared to the sand mould (GSC) resulted in the formation of a finer eutectic. The phase composition was similar to that of the sample cast in the sand mould. In addition to the matrix consisting of a solid solution of α(Al)—points 1 and 6 in Figure 2—a eutectic (α(Al) + Si) was observed in the structure—points 2, 3, 7 and 8 in Figure 2. 2—and secondary particles of α-AlFeMnSi, i.e., Al_15_(Fe, Mn)_3_Si_2_—point 9 in Figure 2—or α-(Al, Mg)FeMnSi, i.e., (Al, Mg)_15_(Fe, Mn)_3_Si_2_—point 4 in Figure 2. The magnesium present in this phase has probably replaced some of the original atoms. The last phase identified in the gravity die-cast (GDC) sample was the δ-phase, i.e., Al_4_(Fe, Mn)Si_2_—see points 5 and 10 in Figure 2. The identification of all phases present by EDX analysis is summarised in Table 3. Figure 2 also shows that the secondary particles were more evenly distributed along the boundaries of the eutectic regions after heat treatment.

**Figure 3:**
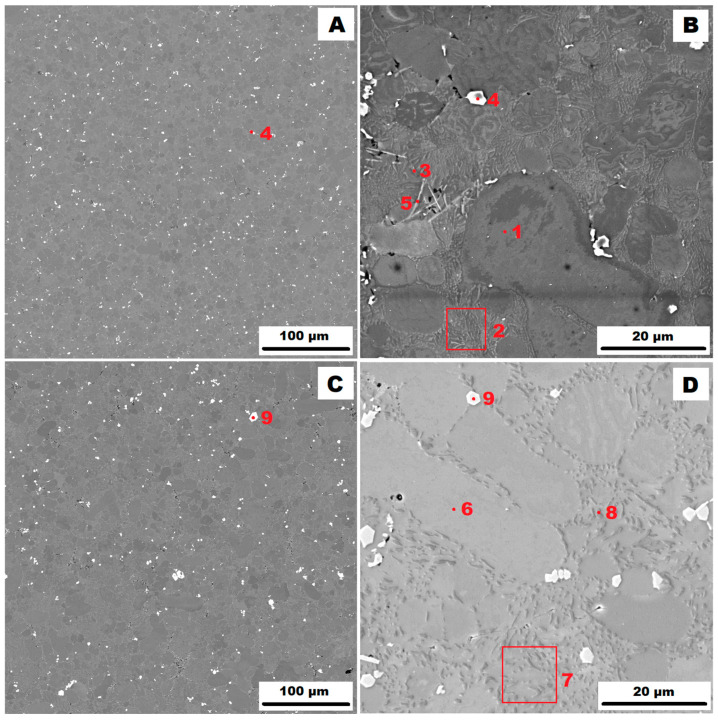
Structure of high-pressure die-casting (HPDC) AlSi10MnMg alloy: (**A**)—as-cast condition (HV 10 kV, BSE); (**B**)—as-cast condition (HV 10 kV, SE); (**C**)—condition after HT400 (HV 10 kV, BSE); and (**D**)—condition after HT400 (HV 10 kV, SE).

The structure of the AlSi10MnMg alloy cast by high-pressure die casting (HPDC) in a vacuum mould is shown in Figure 3 (Figure 3A,B in the as-cast state and Figure 3C,D in the state after heat treatment at 400 °C for 1 h (HT400)). The identification of all phases present by EDX analysis is summarised in Table 4. A comparison of the as-cast states of the studied samples (Figure 1B vs. Figure 2B vs. Figure 3B) shows that the finest eutectic is formed at the highest heat dissipation rate (HPDC). The high heat dissipation rate also affected the phase composition and the shape and distribution of the secondary particles. The EDX analyses showed that in the as-cast state, the unfavourable δ-phase is very rare in the form of small needles in the as-cast sample (HPDC) (see point 5 in Figure 3). After heat treatment at 400 °C for 1 h (HT400), even the presence of the δ-phase was not detected. The precipitates present in the matrix in the as-cast and heat-treated states were identified as α-AlFeMnSi, i.e., Al_15_(Fe, Mn)_3_Si_2—_points 4 and 9 in Figure 3. They were present in the alloy as small, rounded particles and their distribution was very regular.

**Figure 4:**
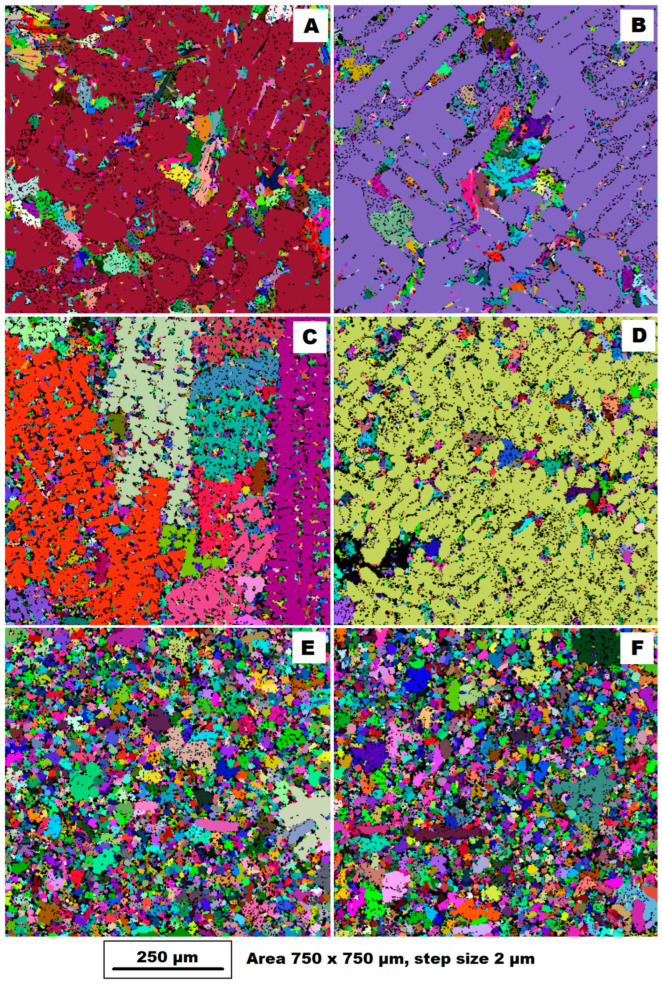
Four EBSD grain maps of investigated alloys (measured area 750 × 750 µm, step size 2 µm, and HV 10 kV): (**A**) gravity sand-cast sample (GSC)—as-cast condition; (**B**) gravity sand-cast sample (GSC)—condition after HT400; (**C**) gravity die-cast sample (GDC)—as-cast condition; (**D**) gravity die-cast sample (GDC)—condition after HT400; (**E**) vacuum high-pressure die casting (HPDC)—as-cast condition; and (**F**) vacuum high-pressure die-casting (HPDC)—condition after HT400.

Figure 4 shows the EBSD analysis of the grains of the studied alloys. From Figure 4A (as-cast condition) and Figure 4B (heat treatment HT400 condition), it can be seen that dendritic grains with random orientation are formed during the slow cooling that occurs during sand casting (GSC). Between the dendrites, there are areas where small grains nucleate. A similar condition can be observed during the solidification of a gravity die-cast (GDC) sample, see Figure 4C—as-cast condition—and Figure 4D—heat treatment HT400 condition). Dendrite growth can be observed in the heat dissipation direction for the gravity die-cast (GDC) alloy in the as-cast state (Figure 4C). The alloy cast by high-pressure die casting into a vacuum mould (HPDC) exhibited a fine-grained structure with regular equiaxed grains (see Figure 4E for the as-cast condition and Figure 4F for the HT400 condition). The grain size and shape remained unchanged after heat treatment.

### 3.2. Effect of Casting Technology on Thermal Diffusivity

**Figure 5:**
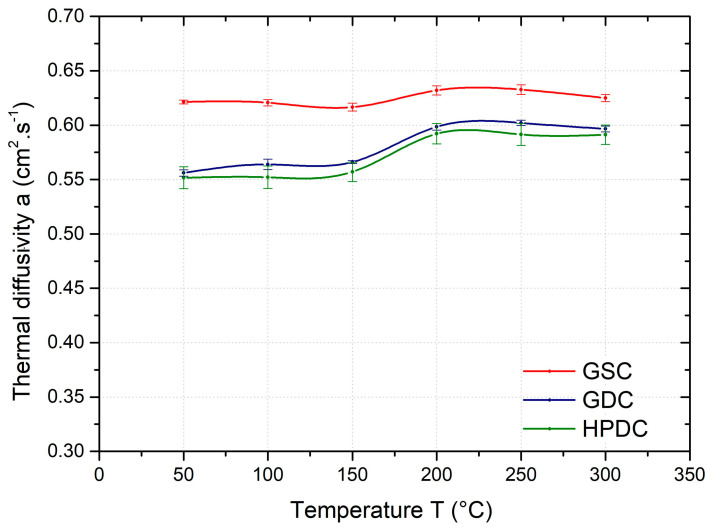
Temperature dependence of average thermal diffusivity of GSC, GDC, and HPDC samples.

Figure 5 shows a comparison of the mean value of the thermal diffusivity coefficient of samples produced by gravity casting in sand and permanent moulds and samples produced by high-pressure die casting as a function of temperature (50–300 °C). The thermal diffusivity of the AlSi10MnMg alloy at 50 °C varies from about 0.55 to 0.62 cm^2^.s^−1^ depending on the casting method. From the results, it can be seen that the thermal diffusivity is highest for the GSC samples. The lower values of thermal diffusivity are achieved by the GDC samples. These differences are due to the different structures of the alloy depending on the casting method. The thermal diffusivity as a function of temperature (50, 100, 150, 200, 250 and 300 °C) shows a slight increase for all samples regardless of the casting method, with a significant change in the temperature range of 150–200 °C.

At 50 °C, the thermal diffusivity of the GSC samples is about 12% higher than that of the HPDC samples; the difference decreases with increasing temperature and is only about 5% at 300 °C. The differences in thermal diffusivity between the HPDC and GDC samples are insignificant from a practical point of view.

### 3.3. Effect of Heat Treatment on Thermal Diffusivity

**Figure 6:**
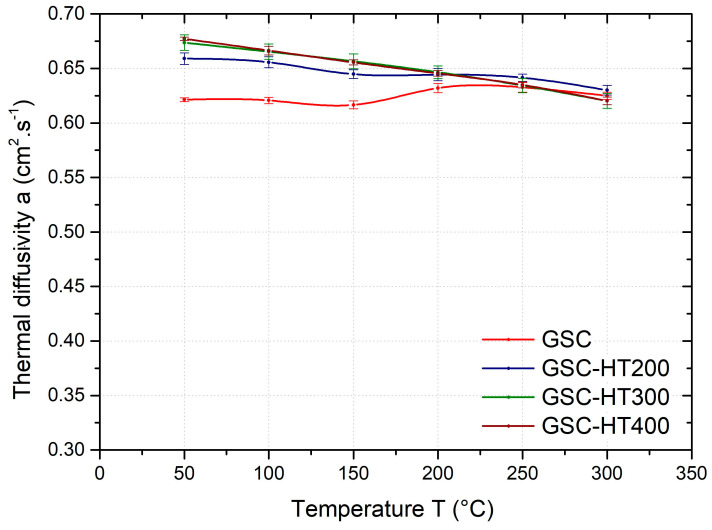
Temperature dependence of the average thermal diffusivity of GSC samples heat-treated at 200 °C (HT200), 300 °C (HT300), and 400 °C (HT400).

**Figure 7:**
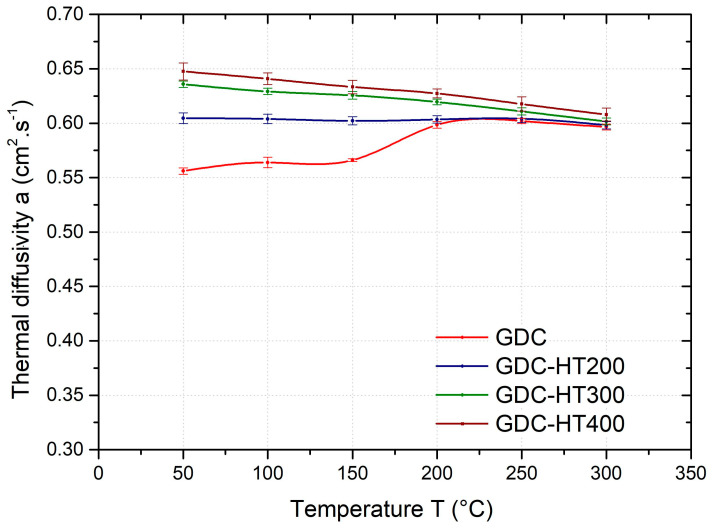
Temperature dependence of the average thermal diffusivity of GDC samples in as-cast condition and heat-treated at 200 °C (HT200), 300 °C (HT300), and 400 °C (HT400).

**Figure 8:**
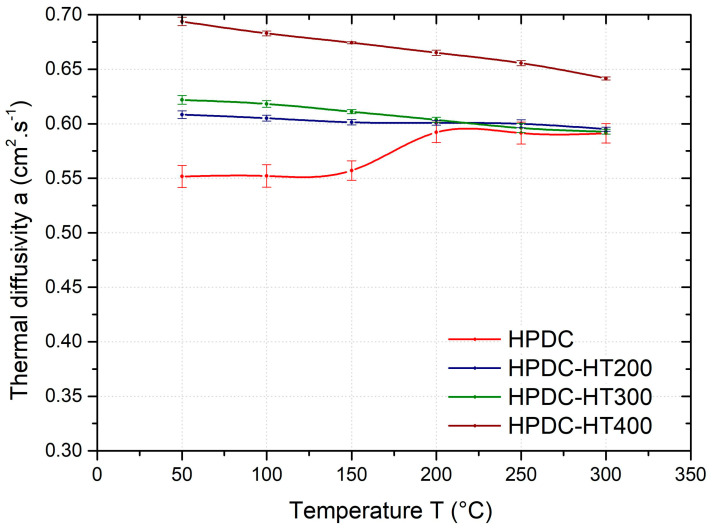
Temperature dependence of the average thermal diffusivity of HPDC samples as-cast and heat-treated at 200 °C (HT200), 300 °C (HT300), and 400 °C (HT400).

The effect of heat treatment on the temperature dependence of the average thermal diffusivity of the GSC samples is shown in Figure 6, GDC samples in Figure 7, and HPDC samples in Figure 8. As can be seen from these figures, the temperature dependence of the thermal diffusivity of samples prepared by different casting technologies and heat treated at 200, 300, and 400 °C (HT200, HT300, and HT400) show the same trends. For all samples (gravity- and die-cast) at temperatures in the range of 50–200 °C, heat treatment (HT200, HT300, and HT400) results in a significant increase in thermal diffusivity. The temperature dependence of the thermal diffusivity also shows no step change after heat treatment at 200 °C and above. In industrial practice, depending on the mechanical properties required, an artificial ageing temperature of 160–230 °C is recommended for this alloy.

Artificial ageing of the GSC samples at 200 °C (HT200) results in a 6% increase in thermal diffusivity at 50 °C; with increasing temperature, the effect of heat treatment diminishes and in the temperature range 200–300 °C the thermal diffusivity is almost the same as in the as-cast condition. Heat treatment at 300 °C (HT300) and 400 °C (HT400) resulted in an increase in diffusivity of approximately 8% (at 50 °C) compared to the as-cast condition. The thermal diffusivity at 300 °C for the HT300 and HT400 heat-treated samples was the same as for the as-cast samples.

Similar curves of the thermal diffusivity versus the temperature are obtained for the GDC samples (Figure 7). Artificial ageing of the GSC samples at 200 °C (HT200) results in an 8% increase in thermal diffusivity at 50 °C; with increasing temperature, the effect of heat treatment diminishes and in the temperature range 200–300 °C the thermal diffusivity is almost the same as in the as-cast condition. Heat treatment at 300 °C (HT300) and 400 °C (HT400) resulted in an increase in diffusivity of approximately 14% and 16% (at 50 °C) compared to the as-cast condition. In the temperature range of 50–200 °C, the heat-treated samples show a slight decrease in thermal diffusivity and at 300 °C the thermal diffusivity reaches the values of the as-cast condition, which, however, shows a rapid increase between 150 and 200 °C.

For the HPDC samples, the temperature dependence of the diffusivity shows a similar trend (Figure 8). After heat treatment at 200 °C (HT200) and 300 °C (HT300), the thermal diffusivity at 50 °C increases by about 10% and 12%, respectively, compared to the as-cast condition. When heat-treated at 400 °C (HT400), the thermal diffusivity value at 50 °C increases by approximately 25%.

## 4. Discussion

A comparison of the thermal diffusivity values of the samples shows that it is influenced by the casting technology (GSC, GDC, and HPDC) and the subsequent heat treatment (HT).

The casting technology results in different cooling rates of the castings and therefore different final structures after casting. Of these technologies, the highest cooling rate is achieved with high-pressure die casting (HPDC). From Figure 4E, it can be seen that HPDC results in a fine-grained structure with regular equiaxed grains. Gravity casting (GC) results in a dendritic structure. In the slow cooling process (GSC), the dendritic grains are randomly oriented, see Figure 4A. As the cooling rate increases, the dendrites grow in the direction of heat dissipation (GDC), see Figure 4C.

**Figure 9:**
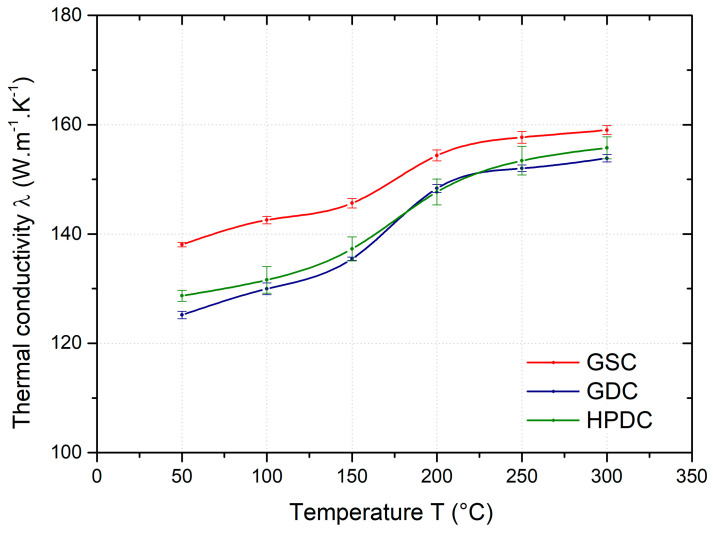
Temperature dependence of thermal conductivity of GSC, GDC, and HPDC samples.

Based on the experimentally determined values of thermal diffusivity, the values of the thermal conductivity coefficient λ were calculated. In the literature [3], the value of thermal conductivity of pure aluminium is reported to be 237 [W·m^−1^·K^−1^]. Alloying elements reduce this value, whether they are dissolved in the solid solution or precipitated as a secondary phase [3,5]. The thermal conductivity of the AlSi10MnMg alloy at 50 °C was found to be in the range of 125 to 138 [W·m^−1^·K^−1^] depending on the casting process. A comparison of the temperature dependence of the thermal conductivity of individual samples, see Figure 9, shows that the thermal conductivity increases slightly with increasing temperature, with a step change at the range of 150–200 °C. In agreement with the results of [8], the highest thermal conductivity values were found for the samples cast in a sand mould (GSC). The thermal conductivity of the GSC samples was approximately 138 [W·m^−1^·K^−1^] at 50 °C, and this value increased to 159 [W·m^−1^·K^−1^] at 300 °C. The thermal conductivity of the HPDC samples was 128 [W·m^−1^·K^−1^] at 50 °C, and approximately 155 [W·m^−1^·K^−1^] at 300 °C. The thermal conductivity of the GDC samples was comparable to that of the HPDC samples. The differences in the thermal conductivity of the GSC and HPDC samples are due to the differences in the amount of dissolved alloying elements in the solid solution, size of particles in the eutectic area, and the different arrangement of secondary phases in the structure of each sample. Similar conclusions were also reported by the authors of [3,5,6].

**Figure 10:**
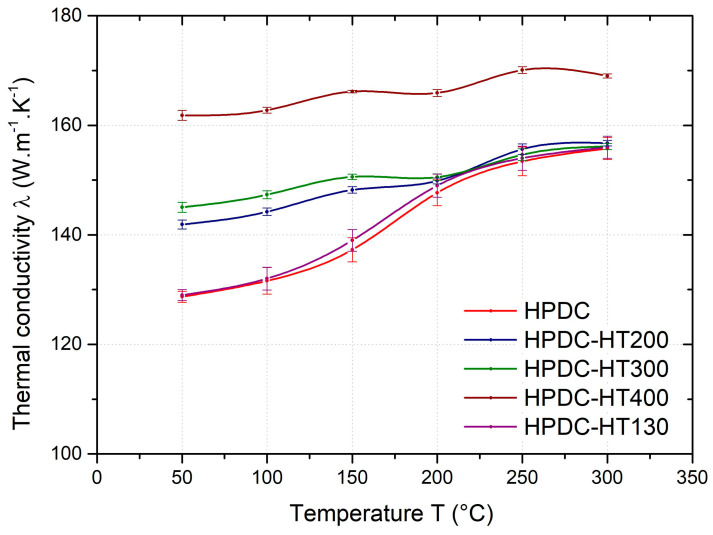
Thermal conductivity of HPDC samples in as-cast condition and heat-treated at 130 °C, 200 °C, 300 °C, and 400 °C.

The rapid increase in thermal conductivity in the temperature range of 150–200 °C for Al-Si-based hardenable alloys can be explained by the change in structure caused by the precipitation of intermetallic phases. According to the authors of [3], alloying elements dissolved in a solid solution have a more significant adverse effect on thermal conductivity than fine precipitates. In Figure 10, the thermal conductivity values for the HPDC samples in the as-cast condition and heat-treated at 130, 200, 300, and 400 °C (HT130, HT200, HT300, and HT400) are plotted as a function of temperature. It can be seen that heat treatment temperatures lower than the reported range of 150–200 °C do not affect the precipitation of the secondary phases and hence the thermal conductivity.

For clarity, Table 5 shows the thermal conductivities of the samples produced by each technology (GSC, GDC, and HPDC) measured at 50 and 300 °C. The values are presented for the as-cast condition as well as the heat-treated condition (HT200, HT300, and HT400).

The experimental results show that even artificial ageing without prior solution treatment can have a positive effect on the thermal conductivity of the material. Regardless of the casting method, an increase in thermal diffusivity or thermal conductivity occurs after heat treatment. In industrial practice, an artificial ageing temperature of about 160–230 °C is recommended for the AlSi10MnMg alloy to increase mechanical properties; higher temperatures lead to overageing and therefore a reduction in mechanical properties. It can be assumed that the recommended precipitation hardening temperatures for increasing the mechanical properties will also have a beneficial effect on the thermal conductivity.

For the GSC samples, artificial ageing at 200 °C (HT200) increases the thermal conductivity at 50 °C by about 6%. Although higher heat treatment temperatures (300 and 400 °C) lead to a slight increase in conductivity due to the refinement of the eutectic silicon (see Figure 1), it can be assumed that the structural changes would lead to a significant decrease in mechanical properties compared to artificial ageing at 200 °C.

For the samples with higher cooling rates (GDC and HPDC), the increase in conductivity after heat treatment is mainly due to the reduction in alloying elements in the solid solution. There is also a refinement of the eutectic and a more uniform distribution of the secondary phases resulting from the decomposition of the supersaturated solid solution, see Figure 2 and Figure 3. The HPDC samples have the lowest thermal conductivity in the as-cast condition and heat treatment has the greatest effect on their thermal conductivity, see Table 5.

## 5. Conclusions

This paper investigates the effect of casting technology (GSC, GDC, and HPDC) and subsequent heat treatment on the thermal conductivity of an AlSi10MnMg alloy. The thermal conductivity was monitored as a function of temperature in the range of 50–300 °C.

The following conclusions can be drawn from the experiments:

- The alloy’s thermal diffusivity or thermal conductivity depends on the casting technology and heat treatment without prior solution treatment. The slower the cooling rate of the casting, the higher the thermal conductivity value. Regardless of the casting method, the thermal conductivity of the samples increases with increasing heat treatment temperature. The alloy prepared by HPDC shows the most significant increase in thermal conductivity.
- The thermal conductivity of the investigated alloys without heat treatment ranges from approximately 125 to 138 [W·m^−1^·K^−1^] at 50 °C and shows an increasing trend as a function of temperature (50–300 °C). The step increase in the temperature range of 150–200 °C is due to the precipitation of intermetallic phases from the solid solution. It is most significant in HPDC, where the cooling rate creates the most supersaturated solid solution.
- The thermal conductivity of the alloy after heat treatment (HT200, HT300, and HT400) shows a similar increasing trend in thermal conductivity depending on the temperature (50–300 °C), but there is no step between 150 and 200 °C. Moreover, the performed research showed that artificial ageing to improve mechanical properties, commonly implemented in industrial practice in the temperature range of 160–230 °C, results in an increase in thermal conductivity. The results also showed that for parts produced by GDC and especially HPDC, where thermal conductivity is important and high mechanical properties are not required, thermal conductivity can be improved by heat treatment at temperatures of 300–400 °C, where blistering does not occur.

## Figures and Tables

**Table 1:** Chemical composition of AlSi10MnMg alloy.

Wt. %	Si	Fe	Cu	Mn	Mg	Cr	Ni	Zn
	10.4	0.115	0.005	0.577	0.273	0.005	0.006	0.005
Wt. %	Pb	Sn	Ti	Na	Sr	Zr	Sb	Al
	<0.003	<0.002	0.063	<0.0005	0.006	0.0027	<0.007	88.50

**Table 2:** Identification of phases from Figure 1.

Point	Phase	Average Chemical Composition [At. %]				
		Al	Mn	Si	Fe	Mg
1	matrix α(Al)	98.5	-	1.5	-	-
2	eutectic (α(Al) + Si)				-	-
3	eutectic particle	1.6	-	98.4	-	-
4	α–(Al, Mg)FeMnSi Al_15_(Fe,Mn)_3_Si_2_	69.2	14.9	12.6	3.3	-
5	δ-phase Al_4_(Fe,Mn)Si_2_	52.3	14.0	30.7	3.0	-
6	matrix α(Al)	98.4	-	1.6	-	-
7	eutectic (α(Al) + Si)					
8	eutectic particle	3.6	-	96.4	-	-
9	α–AlFeMnSi Al_15_(Fe,Mn)_3_Si_2_	69.4	14.7	12.3	3.6	-
10	δ-phase Al_4_(Fe,Mn)Si_2_	53.3	13.5	30.3	2.7	-

**Table 3:** Identification of phases from Figure 2.

Point	Phase	Average Chemical Composition [At. %]				
		Al	Mn	Si	Fe	Mg
1	matrix α(Al)	98.2	-	1.8	-	-
2	eutectic (α(Al) + Si)					
3	eutectic particle	3.1	-	96.9	-	-
4	α–(Al, Mg)FeMnSi Al_15_(Fe,Mn)_3_Si_2_	68.7	13.9	12.3	3.7	1.4
5	δ-phase Al_4_(Fe,Mn)Si_2_	58.9	12.8	25.3	3.0	-
6	matrix α(Al)	98.5	-	1.5	-	-
7	eutectic (α(Al) + Si)					
8	eutectic particle	2.8	-	97.8	-	-
9	α–AlFeMnSi Al_15_(Fe,Mn)_3_Si_2_	76.7	11.8	8.9	2.6	-
10	δ-phase Al_4_(Fe,Mn)Si_2_	54.9	13.1	28.9	3.1	-

**Table 4:** Identification of phases from Figure 3.

Point	Phase	Average Chemical Composition [At. %]				
		Al	Mn	Si	Fe	Mg
1	matrix α(Al)	98.4	-	1.6	-	-
2	eutectic (α(Al) + Si)					
3	eutectic particle	3.2	-	96.8	-	-
4	α–(Al, Mg)FeMnSi Al_15_(Fe,Mn)_3_Si_2_	69.5	15.4	12.7	2.4	-
5	δ-phase Al_4_(Fe,Mn)Si_2_	63.6	9.1	24.5	2.7	-
6	matrix α(Al)	98.5	-	1.5	-	-
7	eutectic (α(Al) + Si)					
8	eutectic particle	3.4	-	96.6	-	-
9	α–AlFeMnSi Al_15_(Fe,Mn)_3_Si_2_	70.0	15.0	12.7	2.3	-

**Table 5:** Average thermal conductivities of individual samples.

Sample	As Cast		HT200		HT300		HT400	
	50 °C	300 °C	50 °C	300 °C	50 °C	300 °C	50 °C	300 °C
	Thermal Conductivity Coefficient λ [W·m^−1^·K^−1^]							
GSC	138 ± 0.4	159 ± 0.8	146 ± 1.0	160 ± 1.0	150 ± 1.6	158 ± 1.7	150 ± 0.4	158 ± 0.9
GDC	125 ± 0.7	154 ± 0.7	136 ± 1.0	154 ± 0.8	143 ± 0.6	155 ± 0.8	150 ± 1.8	157 ± 1.5
HPDC	129 ± 1.0	156 ± 1.9	142 ± 0.8	157 ± 0.5	145 ± 0.9	156 ± 0.6	162 ± 0.9	169 ± 0.4

## Data Availability

The raw/processed data required to reproduce these findings cannot be shared at this time due to legal or ethical reasons.

## References

[1] Biswas P., Patra S., Roy H., Tiwary C.S., Paliwal M., Mondal M.K. (2021). Effect of Mn Addition on the Mechanical Properties of Al–12.6Si Alloy: Role of Al_15_(MnFe)_3_Si_2_ Intermetallic and Microstructure Modification. Met. Mater. Int..

[2] Mrówka-Nowotnik G., Sieniawski J., Wierzbiński M. (2007). Intermetallic phase particles in 6082 aluminium alloy. Arch. Mater. Sci. Eng..

[3] Gan J., Du J., Wen C., Zhang G., Shi M., Yuan Z. (2022). The Effect of Fe Content on the Solidification Pathway, Microstructure and Thermal Conductivity of Hypoeutectic Al-Si Alloys. Int. J. Met..

[4] Radetić T., Popović M., Novaković M., Rajić V., Romhanji E. (2023). Identification of Fe-bearing phases in the as-cast microstructure of AA6026 alloy and their evolution during homogenization treatment. J. Min. Metall. Sect. B Metall..

[5] Zhang A., Li Y. (2023). Thermal conductivity of aluminium alloys—A review. Materials.

[6] Madelung O., Klemens P.G. (1991). Thermal Conductivity of Pure Metals and Alloys.

[7] Davis J.R. (2001). Aluminum and Aluminum Alloys.

[8] Vandersluis E., Lombardi A., Ravindran C., Bois-Brochu A., Chiesa F., MacKay R. (2015). Factors Influencing Thermal Conductivity and Mechanical Properties in 319 Al Alloy Cylinder Heads. Mater. Sci. Eng. A.

[9] Chen J.K., Hung H.Y., Wang C.F., Tang N.K. (2015). Thermal and Electrical Conductivity in Al-Si/Cu/Fe/Mg Binary and Ternary Al Alloys. J. Mater. Sci..

[10] Kim C.W., Kim Y.C., Kim J.H., Cho J.I., Oh M.S. (2018). Effect of Alloying Elements on the Thermal Conductivity and Casting Characteristics of Aluminum Alloys in High-Pressure Die Casting. Korean J. Met. Mater..

[11] Gan J.Q., Huang Y.J., Cheng W.E.N., Jun D.U. (2020). Effect of Sr modification on microstructure and thermal conductivity of hypoeutectic Al-Si alloys. Trans. Nonferrous Met. Soc. China.

[12] Choi S.W., Cho H.S., Kumai S. (2016). Effect of the Precipitation of Secondary Phases on the Thermal Diffusivity and Thermal Conductivity of Al-4.5Cu Alloy. J. Alloys Compd..

[13] Zhou Y., Zhang X., Zhong G., Zhang J., Yang Y., Kang D., Li H., Jie W., Schumacher P., Li J. (2022). Elucidating Thermal Conductivity Mechanism of Al-9Si Based Alloys with Trace Transition Elements (Mn, Cr, V). J. Alloys Compd..

[14] Choi S.W., Kim Y.M., Kim Y.C. (2019). Influence of Precipitation on Thermal Diffusivity of Al-6Si-0.4Mg-0.9Cu-(Ti) Alloys. J. Alloys Compd..

[15] Luo G., Zhou X., Li C.B., Du J., Huang Z.H. (2022). A Quantitative Study on the Interaction Between Silicon Content and Heat Treatment on Thermal Conductivity of Al-Si Binary Alloys. Int. J. Met..

[16] Zhang X., Zhou Y.L., Zhong G., Zhang J.C., Chen Y.N., Jie W.Q., Schumacher P., Li J.H. (2022). Effects of Si and Sr Elements on Solidification Microstructure and Thermal Conductivity of Al–Si-Based Alloys. J. Mater. Sci..

[17] Weng W.P., Nagaumi H., Sheng X.D., Fan W.Z., Chen X.C., Wang X.N. (2019). Influence of Silicon Phase Particles on the Thermal Conductivity of Al-Si Alloys. Light Metals 2019.

[18] Zhang A.L., Li Y.X. (2023). Effect of Alloying Elements on Thermal Conductivity of Aluminum. J. Mater. Res..

[19] Wen C., Gan J.Q., Li C.B., Huang Y.J., Du J. (2021). Comparative Study on Relationship Between Modification of Si Phase and Thermal Conductivity of Al–7Si Alloy Modified by Sr/RE/B/Sb Elements. Int. J. Met..

[20] Wang K., Li W.F., Xu W.Z., Hou S.Y., Hu S.D. (2021). Simultaneous Improvement of Thermal Conductivity and Strength for Commercial A356 Alloy Using Strontium Modification Process. Met. Mater. Int..

[21] Butler C., Babu S., Lundy R., Meehan R.R., Punch J.E.F.F., Jeffers N. (2021). Effects of Processing Parameters and Heat Treatment on Thermal Conductivity of Additively Manufactured AlSi10Mg by Selective Laser Melting. Mater. Charact..

[22] Kim M.S. (2021). Effects of Processing Parameters of Selective Laser Melting Process on Thermal Conductivity of AlSi10Mg Alloy. Materials.

[23] Chen J.K., Hung H.Y., Wang C.F., Tang N.K. (2017). Effects of Casting and Heat Treatment Processes on the Thermal Conductivity of an Al-Si-Cu-Fe-Zn Alloy. Int. J. Heat Mass Transf..

[24] Ramirez A.M., Beltrán F.E., Yáñez-Limón J.M., Vorobiev Y.V., Gonzalez-Hernandez J., Hallen J.M. (1999). Effects of porosity on the thermal properties of a 380-aluminum alloy. J. Mater. Res..

[25] Zhang C., Du Y., Liu S.H., Liu S.J., Jie W.Q., Sundman B. (2015). Microstructure and Thermal Conductivity of the As-Cast and Annealed Al–Cu–Mg–Si Alloys in the Temperature Range from 25 _C to 400 _C. Int. J. Thermophys..

[26] Li K., Zhang J., Chen X.L., Yin Y.H., He Y., Zhou Z.Q., Guan R.G. (2020). Microstructure Evolution of Eutectic Si in Al-7Si Binary Alloy by Heat Treatment and Its Effect on Enhancing Thermal Conductivity. J. Mater. Res. Technol..

[27] Kim Y.M., Choi S.W., Kim Y.C., Kang C.S. (2022). Increasing the Thermal Diffusivity of Al-Si–Mg Alloys by Heat Treatment. J. Therm. Anal. Calorim..

[28] Rauta V., Cingi C., Orkas J. (2016). Effect of Annealing and Metallurgical Treatments on Thermal Conductivity of Aluminium Alloys. Int. J. Met..

[29] Lumley R.N., Deeva N., Larsen R., Gembarovic J., Freeman J. (2013). The role of alloy composition and T7 heat treatment in enhancing thermal conductivity of aluminium high pressure diecastings. Metall. Mater. Trans. A.

